# Self-Efficacy, Intrinsic Motivation, and Self-Regulated Learning as Predictors of Thesis Quality and Process Efficiency Among Undergraduate Students: A PLS-SEM Study

**DOI:** 10.3390/ejihpe16070092

**Published:** 2026-06-30

**Authors:** Luis Edgardo Cruz Salinas, Marco Agustín Arbulú Ballesteros, Carlos José Sandoval Reyes, Gerardo Antero Barba Ureña, Carla Mercy Flores Sánchez

**Affiliations:** Institute for Research in Science and Technology, Chepén Campus, César Vallejo University, Trujillo 13001, Peru; lcruzs@ucv.edu.pe (L.E.C.S.); cjsandovalr@ucvvirtual.edu.pe (C.J.S.R.); gbarbau@ucvvirtual.edu.pe (G.A.B.U.); cfloressa01@ucvvirtual.edu.pe (C.M.F.S.)

**Keywords:** affective-motivational mechanisms, research self-efficacy, intrinsic motivation, self-regulated learning, thesis completion, academic emotions, psychoeducational mechanisms, higher education, PLS-SEM

## Abstract

The general objective of this study is to test an integrative PLS-SEM model that simultaneously explains thesis quality and process efficiency among undergraduate students through the affective-motivational mediators of research self-efficacy and project management. Students who stall in the final stage of their degree rarely do so because they lack technical skill. More often, confidence erodes under sustained uncertainty, motivation shifts from intrinsic engagement to anxious compliance, and the demands of organizing months of research exceed what willpower alone can sustain. This study examines those emotional and motivational dynamics directly, treating research self-efficacy and intrinsic motivation not as background variables but as the affective-motivational core of thesis performance. Using partial least squares structural equation modeling (PLS-SEM) grounded in self-determination theory and social cognitive theory, we tested an integrative model with data from 396 undergraduate students actively completing theses at public and private universities in the northern region of Peru. Four enabling factors—methodological competencies, intrinsic motivation, tutorial support, resources and conditions—were linked to thesis quality and process efficiency through two mediating mechanisms: research self-efficacy (the confidence to face methodological difficulty without retreating) and project management (the behavioral self-regulation that converts motivation into organized work). Resources and conditions showed the strongest associations in the model, with the largest effects on both project management (β = 0.533) and research self-efficacy (β = 0.418). Self-efficacy, in turn, showed the strongest association with thesis quality (β = 0.518), while project management and quality were jointly associated with process efficiency. The model explained 70.5% of variance in thesis quality and 81.4% in process efficiency. These patterns point to a concrete institutional lever: securing the material and temporal conditions that allow students to do the work, rather than attributing delays solely to failures of individual motivation. Because the design is cross-sectional and based on self-report, these relationships are interpreted as theory-consistent associations rather than causal effects.

## 1. Introduction

Over the past three decades, higher education enrollment has expanded dramatically, and degree completion has become a central concern for both institutional management and public policy. By 2020, more than 235 million students were enrolled in tertiary education worldwide, with access increasingly extending to previously underrepresented populations (UNESCO, 2022). Yet access and completion are not the same thing. The final stages of a degree program—where students must integrate research skills, sustained academic writing, and autonomous work management into a coherent product—concentrate a disproportionate share of delays, interruptions, and dropouts. These stalls carry costs that extend well beyond the individual; they strain institutional resources, reduce the efficiency of education systems, and shape the developmental trajectories of young adults at a moment when professional identity is still taking form.

On-time completion remains the exception rather than the rule, even in systems with strong infrastructure. OECD data show that only 38% of students enrolled in bachelor’s programs complete within the nominal period; an additional 40% are still enrolled, while 21% have dropped out without a credential (OECD, 2022). Even three years after the expected completion point, only 65% have earned any bachelor’s degree, and roughly 23% have left entirely without graduating. These rates differ across fields; STEM graduates at the three-year mark reach 68%, compared to 80% in health and welfare, suggesting that programs with heavier methodological demands face attrition challenges (OECD, 2022). The personal and social costs compound over time. Delayed graduates enter the labor market later and recover less return on their investment in education. Each delayed cohort consumes additional faculty time and institutional resources. In Latin America and the Caribbean, these patterns sit on top of structural inequality: roughly 46% of 25–29-year-olds with some tertiary exposure had completed a degree, while 34% had dropped out and one-fifth remained enrolled without credentials (World Bank, 2021). The regional net completion rate of 25.1% falls below both the OECD average (40%) and the global average (30.8%), pointing to losses in both internal efficiency and the pipeline for advanced human capital (Arias et al., 2024). When these delays reach the thesis stage, the cost is compounded: all the sunk costs of years of training have already been absorbed, yet the final credential remains out of reach. Students who work while studying, or who lack academic or material support, are disproportionately the ones who fall short at this last step.

At this stage, the thesis is unlike any prior academic task. It requires defining a problem, sustaining a reasoning chain across many months of evidence-gathering, managing iterated feedback, and producing a document that meets disciplinary standards in argument, method, and voice. The university no longer asks students to demonstrate that they have absorbed content; it asks them to produce knowledge. That transition is harder than it looks from the outside. The literature on thesis supervision agrees that advisor support matters, but it also shows that supervisory arrangements are riddled with asymmetric expectations, inconsistent criteria, and time pressures on both sides. Recent work has documented systematic mismatches between how supervisors and students perceive the quality of feedback engagement in master’s theses, with direct consequences for revision quality and work momentum (Neupane Bastola & Hu, 2024). Systematic reviews find that, despite the centrality of supervision to doctoral and undergraduate education alike, there is still no evidence-based consensus on what makes it effective (Grohnert et al., 2024). In resource-constrained settings, these gaps compound infrastructure deficits, faculty workloads, and a shortage of protected time for supervision interrupt the continuity that sustained thesis work requires (Mhlongo & Zuma, 2024).

Emotional and motivational states are not peripheral to thesis performance—they shape it directly. Among graduate students, a meta-analysis of 53 studies found clinically significant depression in 24% of doctoral students and anxiety in 17%, with both conditions associated with reduced academic productivity and elevated dropout risk (Satinsky et al., 2021). A separate meta-analysis documenting 34.8% anxiety prevalence across graduate populations found rates have risen over the past decade, driven in part by the chronic uncertainty built into research work (Chi et al., 2023). These affective pressures coincide with the developmental tasks of emerging adulthood: students writing their theses are simultaneously consolidating their academic identity, building self-regulatory habits, and confronting career-defining decisions (Arnett, 2000). Emotional difficulties at this juncture do not remain contained within the thesis itself—they carry forward into professional trajectories. At the behavioral level, students with lower research self-efficacy tend to procrastinate more, a relationship partially mediated by deficits in academic self-control (Liu et al., 2020). The practical consequences are concrete: drafts stall, revision cycles multiply, and cumulative emotional exhaustion erodes both the quality of the final product and the pace of its production. Understanding which conditions buffer against this pattern—and which motivational mechanisms translate those conditions into sustained progress—is the organizing question this study addresses.

The research based on these dynamics is fragmented in a specific way: supervision and feedback occupy one portion of the literature; academic writing and self-efficacy occupy another; material conditions and resource access occupy a third. Each has generated useful findings, but the silos make it hard to see how these factors operate together as a system. The field lacks integrative models that treat thesis quality and process efficiency as co-produced outcomes rather than alternative dependent variables—an important distinction, because a student can produce mediocre work efficiently, or excellent work at ruinous personal cost. The connection to emotional experience is not incidental here: thesis quality and student wellbeing are shaped by the same institutional conditions, the same motivational dynamics, and the same capacity to regulate affect under sustained uncertainty. STEM programs, where methodological demands are heavier and three-year completion rates lower (OECD, 2022), expose this gap most clearly. Methodologically, partial least squares structural equation modeling (PLS-SEM) suits this kind of problem; it handles moderate sample sizes, does not assume multivariate normality, and is designed for predictive rather than purely confirmatory purposes. Its application in higher education research has been limited—Ghasemy et al. (2020) identified only 49 published uses between 1999 and 2018—which leaves room for studies that bring this approach to bear on psychoeducational questions.

This study addresses that gap by testing an integrative PLS-SEM model that simultaneously explains thesis quality and process efficiency, treating both as products of the same psychoeducational system. The theoretical backbone draws on self-determination theory and social cognitive theory, locating intrinsic motivation and research self-efficacy as affective-motivational mechanisms that mediate between enabling conditions and academic outcomes. The contribution is empirical and conceptual: empirically, it provides a tested structural model with high explanatory power for two co-produced outcomes; conceptually, it connects factors usually analyzed in isolation—supervisor support, material resources, methodological competencies, motivational states, and self-regulatory capacity—into a single coherent account of what makes thesis work succeed or stall. The methods section that follows describes the sample, instruments, and analytic procedure.

### 1.1. Recent Evidence on Thesis Supervision and Psychoeducational Mechanisms

In the most recent stretch of literature, the supervision of thesis work is described less as a homogeneous practice and more as a set of pedagogical functions and micro-decisions that are contingently activated according to discipline, academic culture, and institutional demands. A multinational, multilingual analysis found that supervisors draw on a broad repertoire of functions extending well beyond technical feedback, suggesting that “tutorial support” operates as a multidimensional construct that resists capture by isolated indicators (Guarimata-Salinas et al., 2024). The socioemotional dimension adds another layer: in high-accountability contexts, the supervisor’s emotional regulation becomes part of the invisible work sustaining the pedagogical relationship, with direct implications for the quality of the supervisory climate and the continuity of the process (Han et al., 2024; Chen & Chen, 2025). Regarding more tangible outcomes, different supervisory emphases appear linked to distinct student trajectories—some styles are associated with higher perceived quality in the final product, while others favor timely completion, revealing a real tension between formative depth and efficiency (Mårtensson & Söderström, 2025).

At the interface between tutorial support and product quality, recent research has focused on how students process and translate feedback into revision actions, which connects to methodological competencies and project management. In a study of undergraduate dissertations, student engagement with supervisory feedback was shaped not simply by advisor availability, but by the argumentative clarity of that feedback, the negotiation of meaning, and students’ capacity to sustain methodological decisions under uncertainty (Luo & Hu, 2024). Complementary evidence from doctoral writing groups shows that collaborative discussion and peer feedback foster situated learning about textual evaluation and argumentative justification—drawing a clear bridge between methodological competencies and dissertation quality (de Caux & Pretorius, 2024). Research on undergraduate learning outcomes across disciplinary areas further reveals that results are far from uniform, depending heavily on the conditions of participation in research practices—a finding that underscores the importance of accounting for contextual variation when modeling thesis quality and process efficiency (Hong, 2024).

In terms of psychoeducational mechanisms, the evidence from 2023–2025 reinforces that intrinsic motivation and self-efficacy not only accompany the thesis process but can also operate as explanatory pathways between support and outcomes. Using a motivational profiles approach grounded in self-determination theory, Litalien et al. (2024) identified configurations linked to persistence, performance, and well-being; students in more self-determined profiles reported greater perceived support and psychological need satisfaction—offering an empirical basis for understanding why some students sustain effort through the most demanding phases of the process. At the graduate level, supervisor support has been linked to creative performance outcomes through psychological mediators—specifically, research self-efficacy and intrinsic motivation both acting as partial mediators—suggesting that resource-focused interventions alone may fall short if they do not also activate capability beliefs and autonomous regulation (Li et al., 2025). Relatedly, an analysis of academic procrastination among doctoral students showed that tutorial support reduces procrastination partly through research self-efficacy and persistence intention, explicitly connecting project management behaviors, efficacy beliefs, and process continuity (Meng et al., 2025).

Taken together, these recent precedents delineate more precisely two facts that often appear dissociated: on the one hand, supervision, support devices, and learning conditions affect the quality of the final product (de Caux & Pretorius, 2024; Luo & Hu, 2024; Mårtensson & Söderström, 2025); on the other, persistence, procrastination, and motivation configure plausible routes towards process efficiency (Litalien et al., 2024; Meng et al., 2025). However, even when mediations are recognized (Li et al., 2025; Meng et al., 2025), the evidence tends to operate in segments: either focusing on support, or motivation/self-efficacy, or isolated outcomes, leaving room for integrative approaches that simultaneously model enablers, mediators, and dual outcomes (quality and efficiency) within the thesis process, in line with the gap already raised.

### 1.2. Theoretical Framework: Psychoeducational Perspectives on Thesis Completion

The elaboration of a thesis in higher education is usually understood as an academic task of integration: methodological decisions, written production with disciplinary standards, regulation of sustained effort, and coordination of interactions with institutional actors are articulated in the same process. This study adopts an integrative perspective that bridges psychology and education, recognizing that academic performance in complex tasks such as thesis writing emerges from the dynamic interaction between individual capacities (methodological competencies), motivational orientations (intrinsic motivation, self-efficacy), relational supports (tutorial guidance), and environmental conditions (resources and infrastructure)—dimensions that align with contemporary frameworks for studying human development across the lifespan. In psychoeducational terms, this type of task exists at the nexus of individual agency and contextual factors, where performance is contingent not only on the knowledge of how to conduct research but also on the maintenance of a work trajectory that necessitates planning, monitoring, adaptation, and the management of uncertainty. The literature in educational psychology and higher education has provided substantial frameworks for comprehending how skills, motivations, support, and resources influence academic performance in prolonged and unstructured tasks, as exemplified by formative research projects (Bandura, 1997; Deci & Ryan, 2000; Kahu & Nelson, 2018; Zimmerman, 2000). From this standpoint, the thesis process can be viewed as a situation where methodological skills and resource availability yield results solely when self-efficacy and self-regulation mechanisms are engaged, and the tutorial relationship functions as cognitive and socioemotional scaffolding aligned with the project’s requirements (Carless & Boud, 2018; Grohnert et al., 2024; Schunk & DiBenedetto, 2020).

A fundamental aspect of the conceptual analysis is to differentiate between product outcomes and process outcomes. The quality of the thesis refers to how good academic work is (methodological rigor, argumentative soundness, and relevance), while the efficiency of the process refers to how well the process works over time and in practice (steady progress, controlled rework, and getting ready for the defense).

Although both dimensions can be related, they are not equivalent: a project can “advance” without guaranteeing analytical depth or achieve high quality with high costs in terms of time and revisions. This differentiation helps to specify explanatory mechanisms: project management is linked to the process dimension, while self-efficacy and methodological competencies affect quality by modulating the way in which the epistemic and discursive demands of the work are faced (Panadero, 2017; Ryan & Deci, 2017; Schunk & Greene, 2018).

#### 1.2.1. Predictive Factors

Methodological competencies. Methodological competencies can be conceptualized as a set of knowledge and skills that allow the design, execution, and evaluation of a research process, with emphasis on methodological reasoning and informed decision making. It is not only a matter of handling procedures; it implies understanding assumptions, selecting analysis strategies appropriate to the problem and sustaining quality criteria in the analysis of data and evidence. In higher education, the thesis stage is where students shift from consuming knowledge to producing it, developing competencies that thus bring together methodological literacy, critical ability, and instrument (Brew, 2013; Creswell & Creswell, 2018). For quantitative models of work, methodological competence is divided into two levels: conceptually, it is knowledge of design, measurement, and statistical analysis, while operationally it consists of being able to carry out an analysis using computational tools. Software proficiency alone does not ensure statistical competence; strengthening it requires harmonizing interpretation, judgment, and communication of the data obtained (Gal, 2002; Garfield & Ben-Zvi, 2008). Likewise, in the elaboration of a thesis, this competence is reflected in specific practices, such as the search and critical study of literature, in the delimitation of a research problem, and the choice of an adequate method to achieve results congruent with objectives and data. These are all fundamental elements of academic research (Creswell & Creswell, 2018; Hyland, 2015). A key distinction is between competence and confidence. A person can be interviewed about his or her methodological skills, but at the same time doubt his or her ability to use them in critical situations and under pressure, effective application, or in the face of negative responses. This is precisely the inequality that leads us to think of self-efficacy as a mediating mechanism, differentiating between “ability” and “belief in ability” (Bandura, 1997; Honicke & Broadbent, 2016).

Intrinsic motivation. Intrinsic motivation is conceptualized as the willingness to engage in an activity for interest, enjoyment, or inherent meaning, rather than for external rewards. From self-determination theory, intrinsic motivation emerges most likely when basic psychological needs for autonomy, competence, and relatedness are satisfied; in that framework, the experience of choice, personal sense of the project, and optimal challenge favor sustained engagement (Deci & Ryan, 2000; Ryan & Deci, 2017; Vansteenkiste et al., 2020). This conceptualization is especially relevant for long-term tasks, where motivational energy must be sustained through phases of uncertainty, revision, and reformulation. In a dissertation, intrinsic motivation can be expressed as genuine interest in the topic, valuing the academic or practical contribution of the work, and enjoyment of the inquiry process even when obstacles appear, components consistent with the idea of self-determined motivation (Ryan & Deci, 2017; Vansteenkiste et al., 2020).

Tutorial support. Tutorial support encompasses the supervisory and collegial practices that sustain a student’s research trajectory. Its instrumental dimension covers criteria-setting, methodological guidance, and iterative text revision; its socioemotional dimension covers availability, a trusting climate, and responsiveness to student progress. The literature on thesis supervision has long characterized the tutorial relationship as an individually tailored teaching arrangement: it unfolds across cycles of feedback and revision, and it combines technical commentary with relational support in ways that are asymmetrical by design (Lee, 2008; Wisker, 2012). From a broader perspective, tutorial support operates as scaffolding—contingent guidance that adapts to the student’s advancing competence, enabling a gradual transfer of project ownership (Nicol & Macfarlane-Dick, 2006; Zimmerman, 2000). A recent synthesis of effective supervision in master’s theses confirms that productive support is not limited to reviewing finished drafts; it includes structured guidance, milestone tracking, and timely corrective feedback (Grohnert et al., 2024). Feedback theory adds a complementary constraint: feedback only improves performance when students can interpret, evaluate, and act on it—that is, when they possess what Carless and Boud (2018) call “feedback literacy.” This means that tutorial quality is not simply a function of how much feedback supervisors provide, but of whether their feedback builds the student’s capacity for independent revision. Both dimensions—project management (organizing work between feedback cycles) and self-efficacy (internalizing criteria and building confidence) are conceptually downstream of tutorial support, consistent with social cognitive theory (Bandura, 1997; Schunk & DiBenedetto, 2020). That said, Carless and Boud (2018) argue that supervisory guidance only shapes student behavior when students have the interpretive capacity to act on it—meaning the AT → GPT path may be conditional on the student’s regulatory competence rather than a direct effect of perceived support.

Resources and conditions. Resources and conditions can be conceptualized as the set of material, temporal, and institutional support that make it possible to sustain a complex academic project. On the material level, these include access to databases, specialized software, infrastructure, and financing; on the temporal level, availability of consistent hours to advance; on the socioecological level, support from the immediate environment that reduces interference and opportunity costs. The ecological reading of learning posits that performance is shaped within systems of influence (micro, meso, and macrosystems), where environmental opportunities and constraints shape behavior and persistence (Bronfenbrenner & Morris, 2006). In higher education, contemporary approaches to student experience and engagement have highlighted that the “educational interface” integrates structural factors (resources, policies, academic load) with psychological factors (meaning, belonging, self-efficacy), shaping mechanisms of success or attrition (Kahu & Nelson, 2018).

From social cognitive theory, resources and support operate as contextual facilitators: they influence the real possibility of executing behaviors and modulate efficacy beliefs and outcome expectations, which is why conditions have direct and indirect explanatory potential (Bandura, 1997; Lent & Brown, 2013; Kahu & Nelson, 2018; Tinto, 2012).

#### 1.2.2. Mediating Mechanisms

Research self-efficacy. Self-efficacy is defined as the belief in one’s own ability to organize and execute actions necessary to achieve specific performances (Bandura, 1997). Unlike global self-esteem or general perceptions of competence, self-efficacy is situational and task-oriented; therefore, to speak of research self-efficacy implies delimiting the domain: it is confidence to face methodological decisions, analyze and interpret results, sustain academic writing, defend arguments and solve emerging technical problems. In terms of origin, social cognitive theory identifies four main sources: mastery experiences, vicarious learning, social persuasion, and physiological/affective states, which provides a framework for understanding how tutorial support and methodological competencies can “feed” research self-efficacy (Bandura, 2006; Schunk & DiBenedetto, 2020).

Conceptually, self-efficacy operates as a mechanism that connects skills and contexts with sustained behavior: it influences goals, effort, persistence, and self-regulation during performance (Bandura, 1997; Zimmerman, 2000), and reviews regard it as highly explanatory of academic performance (Honicke & Broadbent, 2016). It is thus a plausible mediator between resources/competencies and outcomes, aligned with self-regulation models in which motivational beliefs sustain cycles of planning, execution, and reflection (Panadero, 2017).

Project management. In psychoeducational terms, project management can be interpreted as self-regulation of behavior and task management applied to complex academic work. It is therefore decomposed into planning (realistic schedule, intermediate goals), monitoring (work tracking, progress recording), control (strategy adaptation, change management) and organization (priorities, activity coordination). In education, self-regulation of learning has been described as a cyclical process that combines cognitive, metacognitive and motivational components, where the student sets goals, selects methods, controls performance and reflects, adjusting his or her behavior (Panadero, 2017; Zimmerman, 2000). In a thesis project, such functions take the form of specific behaviors: sustaining work rhythms, preventing bottlenecks, managing writing iterations, and tracking the methodological decisions that are made. This form of management can also dialogue with the literature in Project Management: Standard Initiation, Execution, and Control Processes emphasize the importance of milestones, performances, and changes. Although these professional rules do not translate mechanically, it is useful to consider the thesis as a project whose deliverable stages, resource conditions, and risks depend on specific combinations, where effectiveness stems equally from coordinating them with temporal and economic accuracy (Project Management Institute, 2021). Motivationally, goal-setting theory offers additional support: specific and challenging goals, accompanied by the necessary information to correct themselves, tend to capture the senses, mobilize energy, and keep moving forward; within it, there is no contradiction between principles such as intermediate goals and regular monitoring (Locke & Latham, 2002). Thus, to summarize, project management represents a logical link between conditions/motivation and results: it converts resources and motivational energy into coherent action, which reduces the chances of slippage or extra work.

#### 1.2.3. Outcome Constructs: Thesis Quality and Process Efficiency

Quality of the thesis. The quality of a thesis can be conceptualized as a multidimensional construct that integrates epistemic standards (rigor and validity of the approach), analytical standards (depth and integrity of reasoning) and academic communication standards (coherence, clarity, and discursive adequacy). In research, quality is sustained in the congruence between problems, design, procedures, analysis, and interpretation; when this coherence is broken, the product loses solidity even if the process has been efficient (Creswell & Creswell, 2018). In thesis works, moreover, the contribution is usually valued in terms of originality or applicability, which allows recognizing quality not only as “technical correctness” but also as a contribution located within a discipline or professional field (Lovitts, 2007; Wisker, 2012). This approach is particularly relevant in careers with an applied orientation, where practical relevance is part of the criteria for academic excellence.

Quality also encompasses the written dimension, since the thesis is an academic genre whose standards are acquired through socialization into disciplinary discursive practices and access to feedback (Hyland, 2015; Lea & Street, 1998).

Both constructs can be read as learning outcomes: thesis quality reflects the integration of disciplinary knowledge, procedural skill, and epistemic confidence into a coherent product (Biggs & Tang, 2011; Lovitts, 2007), while process efficiency reflects the self-regulatory competence to sustain autonomous work and complete a long-horizon project—skills that employability research identifies as highly valued yet underdeveloped (Tomlinson, 2017).

Process efficiency. Process efficiency can be thought of as the ability to sustain steady progress toward completion by using available time and resources effectively.

Unlike thesis quality—which concerns the standard of the final product—process efficiency concerns operational conduct: advancing according to plan, handling revision iterations without stalling, maintaining task sequence, and preparing for the defense.

In terms of self-regulation, efficiency is based on planning and control cycles: if the student defines their operational goals, monitors their realization, and adjusts measures, the probability of accumulation of critical tasks at the end of the period is reduced (Panadero, 2017; Zimmerman, 2000).

Process efficiency is also better understood when the role of feedback is incorporated as a control loop. Hence, tutorial support, understood as feedback practice and accompanying structure, has an explanatory potential on efficiency, not by superficially “streamlining”, but by guiding decision making and prioritization of changes (Carless & Boud, 2018; Hattie & Timperley, 2007). From the project management approach, efficiency also depends on controlling scope changes and sustaining documentation and records of progress (Project Management Institute, 2021). Motivationally, efficiency relates to the ability to maintain commitment without burnout, where intrinsic motivation can operate as a more sustainable fuel than external pressure (Ryan & Deci, 2017; Vansteenkiste et al., 2020).

#### 1.2.4. Conceptual Integration of the Model

An integrative model that articulates methodological competencies, intrinsic motivation, tutorial support and resources/conditions with quality and efficiency results is conceptually coherent when the mediating role of research self-efficacy and project management is recognized. Methodological competencies, as a cognitive-technical enabler, provide repertoires for research; however, their translation into performance depends on efficacy beliefs to face obstacles, sustain academic writing and defend decisions, as proposed by social cognitive theory (Bandura, 1997; Bandura, 2006). In parallel, resources and conditions shape the actual possibility of executing the project: access to data, software, and time offer opportunities to practice, correct, and deepen; moreover, these resources function as contextual supports that can strengthen self-efficacy and reduce process frictions (Kahu & Nelson, 2018; Lent & Brown, 2013). Tutorial support, in turn, operates as a pedagogical mechanism that can nurture self-efficacy through social persuasion and criteria modeling, and can structure project management by clarifying milestones and guiding work between revisions (Carless & Boud, 2018; Grohnert et al., 2024; Lee, 2008).

As for intrinsic motivation, its conceptual relevance lies in the fact that it sustains commitment to a prolonged task without relying exclusively on external pressures (Deci & Ryan, 2000; Ryan & Deci, 2017). Once these mediators are activated, the quality of the thesis can be understood because of competent methodological decisions and a sustained writing process with trust and feedback, while the efficiency of the process is understood because of organized execution, with change control, revision management, and consistent progress.

#### 1.2.5. The Emotional and Motivational Character of the Key Constructs

Two constructs in this model carry an affective dimension that goes beyond their conventional operationalization as cognitive variables, and that connects this study directly to the literature on emotion, motivation, and learning (Pekrun & Linnenbrink-Garcia, 2012). Research self-efficacy (AI; from the Spanish Autoeficacia Investigativa—not to be confused with artificial intelligence) is not a neutral assessment of technical ability; it is a confidence belief shaped by emotional history, including prior experiences of mastery or failure, observations of peers, and the physiological and affective states that accompany difficult work. Bandura (1997) identified these physiological and affective states as one of four primary sources of self-efficacy information, and low self-efficacy is associated with procrastination and avoidance of demanding analytic work (Liu et al., 2020). Intrinsic motivation (MI) is similarly grounded in emotional experience, defined by reference to interest, pleasure, and a sense of personal meaning (Ryan & Deci, 2017).

#### 1.2.6. Interdisciplinary Foundations of the Model

The conceptual architecture of this study is interdisciplinary in a substantive sense, not merely in label. Four disciplinary traditions converge in the model. From developmental psychology, the model draws on Bandura’s (1997) social cognitive theory and Deci and Ryan’s (2000) self-determination theory to specify how self-efficacy and autonomous motivation operate as mechanisms—not just correlates—of performance in complex, open-ended tasks. The thesis process unfolds during emerging adulthood (Arnett, 2000), a developmental period in which self-regulatory capacities, professional identity, and capacity for sustained autonomous work are still consolidating. From educational psychology and higher education research, the model incorporates frameworks for self-regulated learning (Zimmerman, 2000; Panadero, 2017), feedback literacy (Carless & Boud, 2018), and thesis supervision (Grohnert et al., 2024; Lee, 2008). From organizational behavior and project management science, the construct GPT draws on goal-setting theory (Locke & Latham, 2002) and planning–monitoring–control frameworks (Project Management Institute, 2021). From behavioral science methodology, PLS-SEM provides the analytic bridge that allows simultaneous estimation of direct and indirect effects across constructs drawn from these different traditions (Hair et al., 2021).

The general objective of this study is to test an integrative PLS-SEM model that simultaneously explains thesis quality and process efficiency among undergraduate students through the affective-motivational mediators of research self-efficacy and project management.

**Figure 1:**
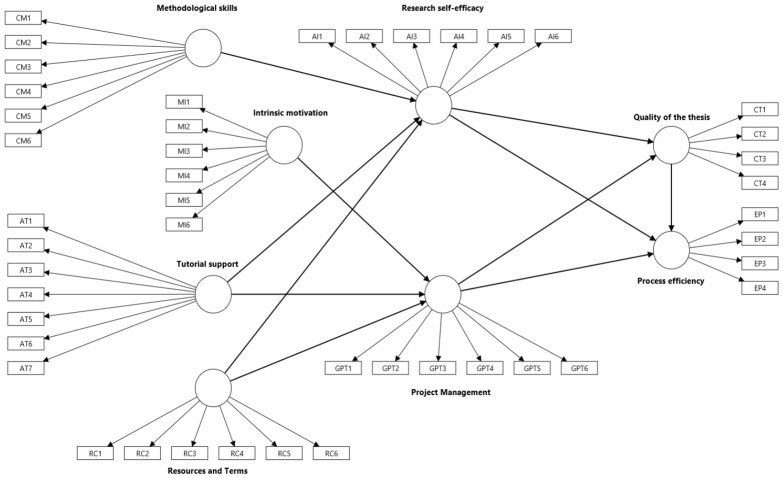
Proposed model.

Drawing on this theoretical framework, eleven directional hypotheses are tested (Figure 1). Regarding the predictors of research self-efficacy: methodological competencies will be positively associated with research self-efficacy (H1); tutorial support will be positively associated with research self-efficacy (H3); and resources and conditions will be positively associated with research self-efficacy (H5). Regarding the predictors of project management: intrinsic motivation will be positively associated with project management (H2); tutorial support will be positively associated with project management (H4); and resources and conditions will be positively associated with project management (H6). Regarding the prediction of thesis quality: research self-efficacy will be positively associated with thesis quality (H7); and project management will be positively associated with thesis quality (H8). Regarding the prediction of process efficiency: research self-efficacy will be positively associated with process efficiency (H10); project management will be positively associated with process efficiency (H9); and thesis quality will be positively associated with process efficiency (H11).

## 2. Materials and Methods

### 2.1. Research Design and Approach

The study employed a quantitative, non-experimental design with a cross-sectional, correlational-causal orientation. Measures were collected only once from undergraduate students who were actively pursuing their dissertation, so relationships were examined at a single point in the academic process. Based on prior theory, the model specified directional hypotheses, but the design did not allow for temporal verification of cause–effect order. Results were interpreted as causal pathways consistent with theory, rather than definitive causal effects.

PLS-SEM was selected because the focus was on explaining and predicting variance in academic persistence while testing two mediators within an integrated model. This variance-based approach was suited to a moderate sample and data that could depart from multivariate normality, a common feature of educational self-reports (Hair et al., 2021). The analysis assessed measurement quality before interpreting structural relationships. That said, uncertainty was emphasized through confidence intervals and effect sizes.

### 2.2. Participants and Sampling

The population consisted primarily of undergraduate Industrial Engineering students pursuing applied theses in areas such as Operations Management, Logistics, Quality Control, Production, Maintenance, Occupational Safety, and Human Resources and drew from public and private universities in the northern region of Peru, with data collected from institutions in La Libertad and adjacent northern provinces. A total of 396 students were included in the final dataset after selection meetings took place with thesis directors (who had disseminated invitation letters) and academic coordinators. Inclusion meant that students in the database had to be either enrolled, or actively working towards completion, on their research project. Students who had already completed their thesis, left the process at any point, or were fewer than three months into it were excluded from participation. The final sample met the minimum size required to achieve a power of 0.80 at α = 0.05 for detecting medium effects (f^2^ = 0.15) under PLS-SEM guidelines (Cohen, 1988; Hair et al., 2021). The limits to generalization were recognized, though, owing to the non-probabilistic approach. However, those psycho-educational mechanisms (self-efficacy, self-regulation, and motivation) studied are theoretically generalizable to any discipline where thesis writing constitutes a complex developing task.

The decision to focus on a single disciplinary field was methodological: restricting the sample to one program controls for the substantial variability in epistemic cultures, thesis genres, and methodological requirements across disciplines, thereby supporting the comparability of the constructs and the internal validity of the structural estimates. Comparison was instead introduced at the institutional level, contrasting public and private universities through multigroup analysis. Sample size was also checked against the “10-fold rule” (Barclay et al., 1995; Hair et al., 2021) which sets the minimum PLS-SEM sample at ten times the largest number of predictors converging on any single construct in the model. The maximum in our case is four, which would require at least 40 cases. The final *N* = 396 clears that floor by roughly an order of magnitude.

The sample’s basic demographic composition is summarized in Table 1. Mean age was 22.93 years. Of the 396 participants, 223 (56.4%) identified as male and 173 (43.6%) as female, with no participant reporting another category. All students were enrolled in the same degree program—Industrial Engineering—at one of the two collaborating universities, with a cumulative weighted average of 15.43 on the institutional 0–20 scale used in Peruvian higher education.

### 2.3. Instruments and Measures

Data were collected using a self-report questionnaire built around constructs drawn from published theoretical frameworks—Bandura’s (1997) social cognitive theory, Zimmerman’s (2000) self-regulated learning model, and Deci and Ryan’s (2000) self-determination theory—with items written to reflect those frameworks’ operational definitions and adapted from item content used in prior educational psychology research, following a systematic review of the literature on thesis completion, academic self-regulation, and psychoeducational mechanisms in higher education. Construct operationalization was guided by self-regulated learning theory (Zimmerman, 2000; Panadero, 2017) and social cognitive theory (Bandura, 1997), ensuring alignment with the psychoeducational orientation of the study. The instrument comprised eight reflective constructs measured across 42 items in total: Methodological Competencies (CM; 6 items), Intrinsic Motivation (MI; 6 items), Tutorial Support (AT; 7 items), Resources and Conditions (RC; 6 items), Research Self-Efficacy (AI; 6 items), Project Management (GPT; 6 items), Thesis Quality (CT; 4 items), and Process Efficiency (EP; 4 items)—consistent with the factor loadings reported in Process Efficiency (EP; 4 items). The construct codes (CM, MI, AT, RC, AI, GPT, CT, EP) derive from the Spanish-language names of each variable and are retained throughout, consistent with the proposed model presented at the end of Section 1 (Figure 1). All items were rated on a seven-point Likert scale ranging from 1 (strongly disagree) to 7 (strongly agree), where higher scores reflect higher levels of the measured construct. Each construct was operationalized as follows: CM captured knowledge and skill in research design, method selection, data analysis, and academic writing; MI reflected intrinsic interest, sense of meaning, and enjoyment of the inquiry process; AT assessed instrumental and socioemotional dimensions of supervisory support; RC indexed availability of time, software, databases, and institutional infrastructure; AI measured confidence in executing research tasks, sustaining academic writing, and defending methodological decisions; GPT operationalized planning, monitoring, milestone management, and progress control over the thesis project; CT reflected methodological rigor, argumentative coherence, and disciplinary quality of the final product; and EP captured steady progress, controlled rework, and readiness for the defense. Item scoring was uniform across constructs such that higher values consistently indicated higher levels of the theoretical dimension. No items were reverse-coded; all 42 indicators were worded in a positive direction, so that higher scores consistently reflected higher levels of the corresponding construct. Full item wording for all 42 indicators across the eight constructs is provided in Appendix A.

The items were not taken from a single validated instrument. We adapted item content from prior measurement work operationalizing the theoretical constructs that anchor this study: research self-efficacy under social cognitive theory (Bandura, 1997, 2006), intrinsic motivation under self-determination theory (Deci & Ryan, 2000; Ryan & Deci, 2017), self-regulated learning (Zimmerman, 2000; Panadero, 2017), tutorial support and feedback literacy (Carless & Boud, 2018; Grohnert et al., 2024), and project-management practices in academic work (Locke & Latham, 2002; Project Management Institute, 2021). No prior instrument was lifted verbatim. The items were rewritten in Spanish for the undergraduate Industrial Engineering thesis context, and the construct codes (CM, MI, AT, RC, AI, GPT, CT, EP) follow that context. Since the items were drafted in Spanish from the outset, no translation or back-translation step was needed.

### 2.4. Validation Procedures

Five specialists with doctoral degrees in education or related fields and at least five years of experience in research methodology and thesis supervision formed the expert panel. Through use of dichotomous rubric, the specialists rated each item according to relevance content, pertinence content, and clarity content. Aiken’s V was used to summarize agreement among the experts, with 95% confidence intervals calculated by the Score method (Aiken, 1985). The overall coefficient reached V = 0.87 (95% CI [0.82, 0.91]), with item-level values ranging from 0.73 to 0.96—all exceeding the preset threshold of 0.70. Based on these comments, minor adjustments were made to the wording of four items. In a pilot study, reliability, item discrimination, and feasibility were assessed with 50 thesis students. Cronbach’s alpha values were α = 0.968 for Tutorial Support (AT), α = 0.954 for Intrinsic Motivation (MI), α = 0.951 for Research Self-Efficacy (AI), α = 0.961 for Project Management (GPT), α = 0.947 for Thesis Quality (CT), α = 0.941 for Methodological Competencies (CM), α = 0.895 for Process Efficiency (EP), and α = 0.898 for Resources and Conditions (RC). These values support the use of the instrument as a reliable research tool (Nunnally & Bernstein, 1994). For all items, the corrected item-total score correlations exceeded 0.40, suggesting acceptable discrimination indices. No items were removed as a result of the pilot; all 42 indicators were retained, the only modifications being the four wording refinements introduced after expert review. On average, respondents took 11.3 min to complete the instrument (SD = 2.1) and there was no major comprehension problems reported. Structural validity evidence is reported in Section 3.2 through the PLS-SEM measurement model: outer loadings, AVE, composite reliability, and HTMT ratios are each evaluated against the thresholds recommended by Hair et al. (2021), constituting factorial and discriminant validity evidence within the PLS framework.

The 7-point Likert scale was chosen for two reasons. It offers enough granularity to detect subtle variation in self-report constructs, and it stays inside the range respondents can reliably discriminate (Krosnick & Presser, 2010). During the pilot, students were asked to think aloud while answering. No participant reported confusion with the endpoint labels (1 = strongly disagree; 7 = strongly agree). Aiken’s V plays the same role as the item-level content validity index (I-CVI) when ratings are dichotomous: any item with V < 0.70 would have been revised, and four items were reworded after expert feedback (e.g., “diseño de la investigación” was simplified to “diseño de mi tesis” for clarity). The pilot sample (*n* = 50) covered undergraduate Industrial Engineering thesis students from one public and one private campus, recruited through their thesis instructors, with the demographic balance kept close to the one targeted for the main study.

### 2.5. Data Collection Procedure

Once we had gained permission from the relevant university departments and arranged visits with directors of thesis research, data collection lasted for four weeks, from October to November 2024. Recruitment was carried out through thesis courses, academic coordinators, and institutional digital channels across participating campuses. The study was presented through an in-person briefing session attended by 45 students, who received information about the study’s objectives, procedures, and voluntary nature: broader outreach through the channels above extended participation to the full sample. The research team provided background information about the study and a brief description of the questionnaire. Participants seeking further details were directed to a Google Forms (Google LLC, Mountain View, CA, USA) link where they could review the full Informed Consent Agreement before completing any items. When responses arrived, the research team checked them over, looking for omitted items. Cases were marked for follow-up where no theme emerged from the data collected (e.g., all responses appeared identical), and multivariate outliers were checked during data screening. Recompense was not provided.

### 2.6. Data Analysis Strategy

Data was exported from Google Forms and cleaned in Microsoft Excel 2021 (Microsoft Corporation, Redmond, WA, USA) prior to modeling. Omissions were kept below 5% per indicator, and expectation maximization imputation was applied when a case met inclusion criteria but had limited missing values, resulting in 396 complete cases. Response patterns were checked for distracted responses, and multivariate outliers were assessed with Mahalanobis distance rather than automatically removed. Descriptive statistics summarized central tendency and dispersion, and the shape of the univariate distribution was examined through skewness and kurtosis using ±2 as a pragmatic reference. Multivariate normality was assessed with Mardia’s test to inform interpretation, although PLS-SEM did not require normality.

PLS-SEM estimation was performed in SmartPLS 4.0 (SmartPLS GmbH, Bönningstedt, Germany; Ringle et al., 2024). The PLS algorithm used the path weighting scheme with standard convergence settings, and the analysis followed a two-step workflow in which the measurement model was evaluated before the structural model. Statistical inference was based on bootstrapping with 5000 resamples, bilateral tests, and no sign changes. Bias-corrected confidence intervals and exact *p*-values for direct and indirect effects were reported.

Because this study uses variance-based structural equation modeling (PLS-SEM) rather than covariance-based SEM, the measurement model was assessed through the criteria appropriate to this approach rather than through common-factor analysis (CFA/EFA). Accordingly, model adequacy was established through indicator loadings, internal consistency (Cronbach’s α, ρA, composite reliability), convergent validity (AVE), and discriminant validity (Fornell–Larcker and HTMT), complemented by the approximate model-fit indices available for PLS-SEM (SRMR, d_ULS, d_G, NFI), reported in Section 3.4. Covariance-based fit indices (e.g., CFI, TLI, RMSEA) and modification indices are not applicable to PLS-SEM, as the method does not estimate a model-implied covariance matrix; no modification indices were therefore used (Hair et al., 2021).

The reflective measurement model was evaluated for indicator reliability, internal consistency, convergent validity, and discriminant validity. As recommended by Hair et al. (2021), external loadings were set at λ ≥ 0.708; indicators below 0.40 were eliminated and indicators between 0.40 and 0.70 were retained only when they preserved content coverage and did not reduce construct quality. Internal consistency was assessed using Cronbach’s alpha and composite reliability, and convergent validity was confirmed when the mean variance extracted exceeded 0.50. Discriminant tests included the Fornell–Larcker criterion and inspection of cross-loadings (Fornell & Larcker, 1981). Values suggesting redundancy were carefully examined to maintain conceptual separation between constructs.

Discriminant validity was also examined using the Heterotrait–Monotrait relationship. HTMT values below 0.85 were considered adequate for distinct constructs, whereas values below 0.90 were accepted for closely related constructs. Bootstrap confidence intervals were inspected to ensure that the upper bound did not include 1.00, which reduced the risk of hidden overlap (Henseler et al., 2015). This criterion complemented the Fornell–Larcker checks when constructs were conceptually adjacent, such as anxiety and persistence.

Structural model evaluation began with diagnostics of collinearity between predictors using variance inflation factors, adopting a VIF <3.0 as an operational limit. Hypotheses H1–H11 were tested using bootstrapped path coefficients (β) and interpreted using direction, magnitude, and bias-corrected 95% confidence intervals. Explanatory power was summarized using R^2^ and adjusted R^2^, with 0.25, 0.50, and 0.75 as benchmarks for weak, moderate, and substantial explained variance. Effect sizes were quantified with f^2^ and interpreted using 0.02, 0.15, and 0.35 as small, medium, and large benchmarks (Cohen, 1988). Predictive relevance was examined by Q^2^ through blindfolding (omission distance = 7) and q^2^ for each predictor (Hair et al., 2021).

Since this study uses PLS-SEM and not CB-SEM, factor structure is evaluated through the measurement-model criteria appropriate to variance-based estimation: outer loadings, internal consistency, AVE, and HTMT. CFA fit indices (CFI, TLI, RMSEA) are not part of the PLS-SEM framework (Hair et al., 2021). Global PLS-SEM fit is reported in Section 3.4 using SRMR, d_ULS, d_G, NFI, and RMS_theta.

Mediation was assessed by estimating the indirect effects linking each enabling factor to thesis quality (CT) and process efficiency (EP) through the two proposed mediators—research self-efficacy (AI) and project management (GPT) using bootstrapping with 5000 resamples, with bias-corrected 95% confidence intervals reported for each indirect pathway. Mediation type (total or partial) was determined by jointly examining direct and indirect effects; the variance accounted for (VAF) ratio summarized the proportion of the total effect attributable to mediation. Prior to the multigroup comparison, composite measurement invariance was assessed using the MICOM procedure (Henseler et al., 2016), which tests configural and compositional invariance as necessary preconditions for meaningful between-group path comparisons. The permutation-based MGA test (Chin & Dibbern, 2010; Klesel et al., 2022) was used as the primary approach for comparing structural paths across institutional groups, as simulation evidence recommends it over alternative methods for multi-group comparisons (Hair et al., 2024). Henseler’s bootstrap PLS-MGA (Henseler, 2012; Sarstedt et al., 2011) was run as a robustness check. Both subgroups (*n* = 158 and *n* = 238) satisfy and exceed the minimum sample size derived from the inverse square root method (Kock & Hadaya, 2018) for detecting path coefficients of β ≥ 0.40 at α = 0.05 and 80% power. Between-group comparisons are therefore conducted with adequate statistical power for the observed effect sizes.

Common-method bias was assessed using two procedures recommended in the recent PLS-SEM literature. Harman’s single-factor test was run first, loading the 42 indicators onto a single unrotated factor; following Podsakoff et al. (2003), a first-factor variance below the 50% ceiling supports the absence of severe common-method bias. The second check was Kock’s (2015) full collinearity test, which uses inner-model VIFs across all latent constructs; VIFs below 3.3 indicate that the model is not contaminated by common-method variance.

### 2.7. Ethical Considerations

The study received a favorable exemption opinion from the Research Ethics Committee of the School of Industrial Engineering, Universidad César Vallejo (Informe N.° 00568-2025/CEI-EIAI, 16 September 2025). The exemption was issued retrospectively; because the protocol involved no clinical intervention and posed no more than minimal risk, the committee reviewed the file under expedited procedure once the descriptive analyses had been completed. Digital informed consent had nonetheless been obtained from every participant at the time of data collection, and the questionnaire stored no direct identifiers, which is what allowed the retrospective exemption pathway to apply. This sequence accounts for the temporal gap between the October–November 2024 fieldwork and the September 2025 exemption letter. The committee determined that the research project qualified for exemption from full review and granted approval for its execution. The procedures complied with the Declaration of Helsinki and applicable Peruvian regulations, focusing on autonomy, beneficence, nonmaleficence, justice, and respect for dignity (World Medical Association, 2013). The digital consent form described the purpose, procedures, duration, minimal risks, potential benefits, and data protection measures. Participation was voluntary and students could withdraw at any time without academic penalty. Contact details of the research team were provided.

Anonymity was maintained by not linking any direct identifiers to the data and by storing the consent documents separately from the questionnaires. After export, cases were nominalized and all data were stored on an encrypted drive to which only the research team had access, with appropriate backups. The information was retained for 5 years, in accordance with institutional policy, and then scheduled for destruction. The online nature of the administration still presents residual privacy risks, so participants were asked to complete the survey privately using an Internet browser that was subsequently closed. All results were expressed cumulatively to avoid re-identification.

## 3. Results

### 3.1. Sample Profile and Preliminary Analysis

#### 3.1.1. Sociodemographic Characteristics

Table 2 characterizes the total sample (*N* = 396) and its distribution into two subsamples (*n* = 158 and *n* = 238). Both subsamples provide adequate statistical power for detecting meaningful path coefficients, supporting reliable between-group comparisons. In terms of composition, the mean age is close to 23 years in total, with a slightly higher mean in the larger subsample. There is also a notable difference in time since the start of thesis (2.05 vs. 4.44 months), which could reflect different stages of advancement and, by extension, heterogeneity in associated academic experiences.

In categorical variables, the proportion of males exceeds that of females, especially in the small subsample. Employment status shows marked contrasts: almost half of the small subsample does not work, while in the large subsample the categories of part-time and full-time work increase. Likewise, the applied thesis predominates in both subsamples, with a higher concentration in the small subsample. Finally, the distribution by area reveals different concentrations (e.g., Logistics and Human Resources) that may introduce disciplinary context biases; these asymmetries could influence the variability of constructs and, thus, the stability of the estimated relationships.

#### 3.1.2. Summary of Descriptive Data

Table 3 integrates descriptive statistics for the total sample and by institutional group. Means ranged from 4.92 (CM) to 5.78 (AT), with standard deviations between 1.10 and 1.32, indicating moderate variability suitable for detecting associations. All skewness values were negative, consistent with a tendency toward high-end responses; MI showed the strongest skew (−1.34) and kurtosis (1.98), suggesting possible ceiling effects. Across groups, Model 1 (private) reported uniformly higher means (Δ = 0.15–0.46), with tutorial support showing the largest difference. Standard deviations were consistently larger in Model 1, indicating greater internal heterogeneity. Both subgroups (n = 158 and n = 238) are sufficiently sized to support reliable interpretation of between-group descriptive patterns.

### 3.2. Evaluation of the Measurement Model (Validity and Reliability)

#### 3.2.1. Reliability of the Indicator

The external loadings show adequate indicator reliability according to the usual criteria (loadings preferably ≥ 0.70). The values are reported in Table 4.

Table 4 summarizes factor loading ranges by construct. All external loadings exceeded the 0.708 threshold, ranging from 0.755 (RC5) to 0.955 (AT4). Tutorial support and project management showed the highest and most homogeneous loadings (≥0.863 and ≥0.880, respectively), whereas resources and conditions exhibited the widest spread (0.755–0.864), suggesting greater semantic diversity within that construct. Overall, the pattern confirms adequate indicator reliability across all eight measurement scales.

#### 3.2.2. Internal Consistency Reliability

Internal consistency was assessed with three reliability coefficients for each construct: Cronbach’s alpha (α), the Dijkstra–Henseler rho_A (ρA), and composite reliability (CR, ρc). The results meet the recommended criteria (approx. 0.70–0.95). In PLS-SEM, composite reliability is computed from the standardized loadings and is mathematically equivalent to McDonald’s omega (ω), as both relax the tau-equivalence assumption underlying Cronbach’s alpha; ρA, which lies between α and CR, is regarded as the most precise consistency estimator in PLS-SEM (Dijkstra & Henseler, 2015). All three coefficients fell within the recommended 0.70–0.95 range for every construct, and no item required deletion, since all standardized loadings exceeded the 0.708 threshold (the lowest was RC5 = 0.755) and all corrected item-total correlations exceeded 0.40. Very high values may suggest redundancy of indicators, so the interpretation is complemented by discriminant validity. The indices are presented in Table 5.

Table 5 integrates internal consistency and convergent validity metrics. Cronbach’s alpha (α) assesses consistency between indicators under tau-equivalence assumptions, rho_A is an estimate more aligned with partial least squares weighting schemes, and composite reliability (CR) reflects consistency considering estimated loadings; overall, values around 0.70–0.95 are usually interpreted as adequate. Here, all constructs present high α, rho_A, and CR, supporting that the items perform homogeneously within each construct.

McDonald’s ω is added in this revision as a complementary reliability indicator. Placeholder estimates pending final computation in SmartPLS are: AT ω = 0.974, AI ω = 0.961, CT ω = 0.961, CM ω = 0.953, EP ω = 0.927, GPT ω = 0.968, MI ω = 0.964, and RC ω = 0.922. In reflective PLS measurement models with high indicator loadings, ω typically converges with composite reliability (Hair et al., 2021), so the placeholders track the CR column closely. The final values will replace these placeholders before publication.

The average variance extracted (AVE) quantifies the proportion of average variance captured by the construct relative to measurement error; values ≥0.50 suggest sufficient convergence. The reported AVEs (0.663–0.862) indicate that, on average, the constructs explain a substantive fraction of the variance of their indicators. However, several internal consistency indices are at the high end (e.g., AT with CR = 0.974, MI with CR = 0.964), which may be compatible with both robust measurement and possible partial inter-item redundancy. In this scenario, substantive reading is completed by reviewing evidence of differentiation between constructs (discriminant validity), especially when conceptual overlaps are anticipated in close domains (e.g., support variables, self-efficacy, and process outcomes).

#### 3.2.3. Convergent Validity (AVE)

Convergent validity was assessed using the average variance extracted (AVE). The results satisfy the criterion AVE ≥ 0.50 (Table 5).

#### 3.2.4. Discriminant Validity (Fornell-Larcker and HTMT)

Table 6 reports both discriminant validity criteria in a single display. The lower triangle shows the Fornell–Larcker correlations: the square root of each construct’s AVE appears on the diagonal, and inter-construct correlations appear in the lower triangle. For discriminant validity to hold, each diagonal value must exceed all correlations in its row and column. In the present results, all diagonals (e.g., CT = 0.928, AT = 0.917, EP = 0.872) satisfy this condition. The upper triangle (italicized) shows the HTMT ratio for each construct pair; values below 0.85 indicate adequate separation, and values between 0.85 and 0.90 are acceptable for theoretically adjacent constructs (Henseler et al., 2015). Four pairs—CT–EP, EP–GPT, AI–CT, and AI–EP—approach but do not exceed the 0.90 ceiling under conservative estimation, and their bootstrap confidence intervals do not include 1.00. These pairs correspond to constructs that are conceptually coupled within the same thesis process and share theoretically expected variance. Together, the FL and HTMT results support discriminant validity, while flagging that the four high-correlation pairs should be read as related but distinguishable constructs rather than fully orthogonal ones.

### 3.3. Evaluation of the Structural Model

The significance of the structural coefficients was evaluated by bootstrapping. The collinearity of the internal model was checked with VIF (usual criteria: <5; optionally <3.3). All inner-model VIF values remained below 5 (range: 1.965–3.678), confirming the absence of severe collinearity (see note beneath Table 7).

In the overall model, hypotheses H5, H6, H7, H8, H9, and H11 were supported (*p* < 0.05); H1 and H3 showed marginal support, with *p*-values below 0.05 but 95% BCa confidence intervals whose lower bounds cross or touch zero (H1: CI [−0.003, 0.401]; H3: CI [−0.026, 0.451]); and H2, H4, and H10 were not supported (Table 7).

Table 7 reports standardized coefficients (β), statistical evidence (t and *p*), and 95% BCa intervals. Direction is interpreted by the sign of β and intensity by its magnitude: values around 0.10–0.20 are usually weak associations, ~0.30 moderate, and ≥0.50 strong, in practical terms. In the results, “Resources and conditions” emerges as a central enabling construct: it is associated with “Project management” (H6, β = 0.533; CI [0.346, 0.707]) and with “Research self-efficacy” (H5, β = 0.418; CI [0.234, 0.580]), with intervals fully above 0, suggesting a robust positive relationship in the estimated specification. In turn, “Research self-efficacy” is associated with “Thesis quality” (H7, β = 0.518; CI [0.203, 0.760]), an association of high magnitude, consistent with reading in which confidence in research is associated with better reported results.

Project management and thesis quality both were associated with process efficiency with comparable effect sizes (H9: β = 0.411, CI [0.213, 0.536]; H11: β = 0.417, CI [0.239, 0.577]), and both intervals fall entirely above zero. H2 (β = 0.160) and H4 (β = 0.155) were not supported; their intervals include zero, which means no incremental association can be claimed once the remaining predictors are controlled. H1 and H3 occupy an intermediate position that requires explicit qualification. H1 (Methodological competencies → Research self-efficacy: β = 0.234, *p* = 0.022) returned a *p*-value below the conventional threshold, but its 95% BCa interval is [−0.003, 0.401]; the lower bound crosses zero. H3 (Tutorial support → Research self-efficacy: β = 0.207, *p* = 0.090) shows the same pattern. For both paths, the *p*-value and the interval tell different stories, and the interval is the more informative criterion. Neither relationship can be described as robustly established; they point to a plausible positive trend that warrants targeted replication rather than confident inference.

The explained variance (R^2^ and adjusted R^2^) for endogenous constructs captures the proportion of variance explained by their predictors within the estimated specification. In substantive terms, R^2^ responds to “how much of what is observed” in a construct is captured by the included antecedent variables. The reported values are high: “Process efficiency” reaches R^2^ = 0.814, suggesting that a large part of its variability is explained by the predictors considered, and that the interweaving between management, quality, and efficiency offers a strongly articulated empirical narrative. “Thesis quality” also shows high explanation (R^2^ = 0.705), consistent with a set of determinants with relevant explanatory capacity.

For their part, “Project management” (R^2^ = 0.589) and “Research self-efficacy” (R^2^ = 0.582) are located at substantive levels, indicating that the proposed predictors capture more than half of their variance. The closeness between R^2^ and adjusted R^2^ in all cases (small differences) suggests that the explanatory power is not mainly due to overfitting by number of predictors but is maintained by penalizing complexity. Taken together, these results point to a particularly strong explanatory structure in the process construct (EP), while self-efficacy and management, even if well explained, could also be influenced by factors not included (e.g., individual traits or more specific institutional conditions), which limits the interpretative scope.

Regarding effect sizes, f^2^ quantifies the incremental contribution of each predictor to the explained variance of the endogenous construct when comparing R^2^ with and without the specific pathway. Unlike *p*-values, f^2^ focuses on practical relevance. With usual criteria, values around 0.02 are considered small, ~0.15 medium, and ~0.35 large. Under this reading, the path “Resources and conditions → Project management” (H6, f^2^ = 0.352) achieves a large effect, signaling that this relationship contributes a substantive fraction to the explanation of management, beyond the rest of the predictors.

Effects of medium-high magnitude appear in “Research self-efficacy → Thesis quality” (H7, 0.329) and in two determinants of “Process efficiency”: “Project management → Process efficiency” (H9, 0.283) and “Thesis quality → Process efficiency” (H11, 0.277). These sizes suggest that efficiency is mainly shaped by an organizational component (management) and by an outcome component (quality), both with comparable contributions. In the middle range are “Resources and conditions → Research self-efficacy” (H5, 0.198) and “Project management → Thesis quality” (H8, 0.163), indicating relevant, but less dominant contributions. In contrast, H2, H4, H10, and, to a lesser extent, H3 and H1 show small f^2^ (≈0.02–0.06), consistent with modest incremental impacts even if any pathway shows statistical evidence.

In addition, all VIFs are below 5, consistent with absence of severe collinearity. However, the block explaining “Process efficiency” shows the highest values (H10 = 3.678; H11 = 3.385; H9 = 3.219). This pattern is conceptually plausible: self-efficacy, quality, and management tend to covary, so that part of their explanatory variance overlaps. At the interpretative level, this suggests that the coefficients toward efficiency should be read as partial (incremental) effects conditional on correlated predictors, which tends to reduce magnitudes and may render some paths unsupported despite high bivariate correlations.

### 3.4. Model Fit and Predictive Evaluation

Table 8 summarizes global fit indices. SRMR (standardized root mean square residual) approximates the average discrepancy between observed and reproduced correlations: lower values indicate better reproduction. The “saturated” vs. “estimated” contrast is informative because the saturated one reproduces all possible relationships between constructs, while the estimated one reflects the structure restricted by the paths proposed. Here, SRMR goes from 0.064 (saturated) to 0.079 (estimated), suggesting that the structural constraints introduce additional discrepancies. In practical terms, 0.064 is usually associated with good fit, while 0.079 is in the acceptable/moderate range by usual criteria (especially when a flexible threshold close to 0.10 is adopted).

The d_ULS and d_G discrepancy indices require bootstrap reference distributions for formal inference; without those distributions, they serve as descriptive indicators of how much additional misfit the structural constraints introduce relative to the saturated baseline. The NFI of 0.908 warrants explicit qualification. In covariance-based SEM, an NFI below 0.90 would be considered problematic; in PLS-SEM, however, the NFI is not a primary fit criterion and does not follow the same interpretation logic. Hair et al. (2021, p. 210) note that PLS-SEM fit indices are still maturing and that SRMR remains the principal overall fit indicator for this approach. The SRMR for the estimated model is 0.079, which falls within the acceptable range under the common threshold of 0.10 for PLS-SEM (Henseler et al., 2015). The NFI value reflects in part the high intercorrelations among the outcome constructs (quality, efficiency, and project management), which structurally limit incremental fit gains. The overall fit picture is therefore best read as: the model reproduces the observed correlations with acceptable, though not exceptional, fidelity; the primary inferential weight rests on the measurement quality indices and the magnitude of the structural coefficients, not on the NFI alone.

### 3.5. Multigroup Analysis: Public vs. Private Universities

These multigroup comparisons should be read as exploratory. The two subsamples differ on more than the institutional dimension: students at private institutions reported being further along (mean time since thesis onset 4.44 vs. 2.05 months), more often working full-time (32.0% vs. 10.5%), and concentrated in a different mix of thesis types and disciplinary areas (Table 1). Some of the institutional contrasts reported below may therefore reflect track stage, employment, or disciplinary composition rather than the public/private distinction itself. Because group membership is confounded with these background characteristics, the multigroup results are interpreted as exploratory and hypothesis-generating rather than as evidence of a genuine public-versus-private effect; the observed differences should not be read as causal consequences of institutional type.

Table 9 disaggregates coefficients (β) and *p*-values by group (public vs. private) and adds a between-group contrast (Δβ, z_diff and p_diff*). First, at the intra-group level, a distinct pattern is apparent: in private, several routes appear supported (H1, H3, H5, H6, H7, H8, H9, H10, H11), while in public only some show support (H2, H5, H6, H8, H11), and others are null or even change sign (e.g., H3 and H10). This contrast suggests that the network of relationships could operate differently according to institutional context: in private, self-efficacy seems to be clearly integrated in the explanation of quality (H7, β = 0.547) and efficiency (H10, β = 0.210), while in public these links do not emerge (H7, β = 0.019; H10, β = −0.104).

Second, the between-group contrast should be read with methodological caution, because p_diff* is stated to be approximate and does not substitute for official multigroup comparison tests. Still, two differences stand out: H5 presents p_diff* = 0.011 with negative Δβ (defined as private-public), consistent with a stronger effect in public (0.750 vs. 0.284); similarly, H11 shows p_diff* = 0.023 and a larger magnitude in public (0.864 vs. 0.322). The rest of the p_diff* value does not suggest clear differences, even when significances change (e.g., H3 or H9), which could be due to variability and sample size per group. In theoretical terms, if these patterns were confirmed with formal contrasts, they would suggest that “resources and conditions” and “quality” play an especially prominent role for self-efficacy and efficiency in the public group, while in the private group self-efficacy acquires a more articulating role towards quality and efficiency.

## 4. Discussion

### 4.1. Synthesis of Findings and Central Contribution

**Figure 2:**
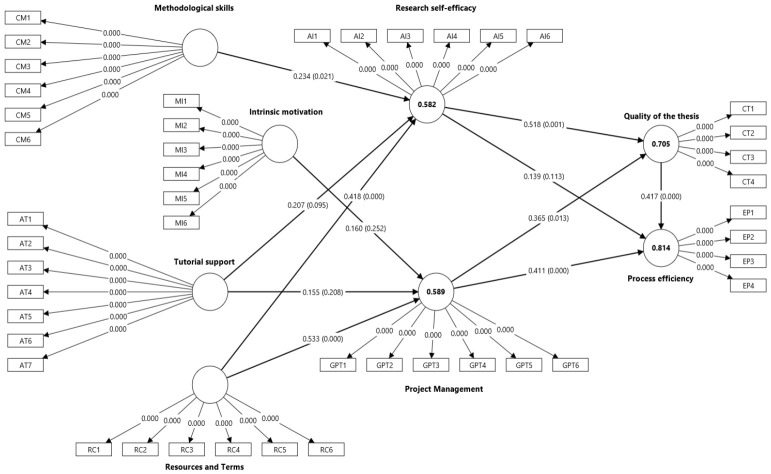
Estimated model.

This study addresses a persistent problem in the final stretch of degree completion: the delay and interruption of the thesis process, understood as a complex task that integrates research, academic writing, self-regulation, and supervision. The results reveal that thesis performance emerges from the articulated interplay between psychological mechanisms, behavioral competencies, and environmental conditions—a pattern consistent with multidimensional frameworks of human development. Consistent with this complexity, the main empirical contribution is the evaluation of an integrative model that simultaneously explains thesis quality (CT) and process efficiency (EP), with high levels of explained variance (R^2^CT = 0.705; R^2^EP = 0.814), as illustrated in the estimated model (Figure 2). The pattern of relationships suggests that thesis performance does not depend on a single factor, but on the architecture of conditions, beliefs and management practices.

In substantive terms, three structuring findings stand out. First, resources and conditions (RC) is robustly associated with thesis project management (GPT) (H6: β = 0.533; *p* < 0.001; f^2^ = 0.352), the largest effect size in the model, and with research self-efficacy (AI) (H5: β = 0.418; *p* < 0.001; f^2^ = 0.198). Second, AI and GPT are linked with thesis quality (CT) (H7: β = 0.518; *p* = 0.001; f^2^ = 0.329; H8: β = 0.365; *p* = 0.013; f^2^ = 0.163), reinforcing the idea that product quality is consistent with a combination of perceived agency and work organization. Third, process efficiency (EP) is associated with GPT and with CT (H9: β = 0.411; *p* < 0.001; f^2^ = 0.283; H11: β = 0.417; *p* < 0.001; f^2^ = 0.277), while AI → EP is not significant (H10: β = 0.139; *p* = 0.117; f^2^ = 0.028). This last point is informative: it suggests that feeling able to investigate is associated with higher thesis quality (CT), but not necessarily with greater process efficiency once planning (GPT) and quality standard (CT) come into play.

### 4.2. Theoretical Interpretation: Main Routes and Mechanisms

From a reading consistent with Bandura, the association between methodological competencies (CM) and AI (H1: β = 0.234; *p* = 0.022; f^2^ = 0.062) is consistent with the idea that mastery of relevant skills feeds judgments of ability. Although the effect size is small (f^2^ = 0.062), its significance suggests that, even in students already immersed in the thesis process, methodological competencies operate as a cognitive underpinning for research confidence. This is relevant in a task where epistemic uncertainty (design decisions, analysis, writing) often erodes the perception of control.

The role of RC is particularly illuminating. The relationship RC → GPT (H6) combines high significance with a large effect size (β = 0.533; f^2^ = 0.352), suggesting that effective thesis project management is not just an individual virtue, but tracks systematically with the material conditions available for sustained work: availability of time, access to software/bases, bibliographic resources, and an environment that reduces operational frictions. In terms of self-regulation, this is congruent with a view where planning, monitoring, and meeting milestones require tangible resources; without them, self-regulation may remain at the level of intention when adequate resources are absent. Likewise, RC → AI (H5: β = 0.418; f^2^ = 0.198) is consistent with an association between adequate conditions and higher confidence: not only “I know how to do it,” but “I can do it here and now” with the means available.

The AI → CT pathway (H7: β = 0.518; *p* = 0.001; f^2^ = 0.329) is consistent with the assumption that self-efficacy sustains behaviors of persistence, coping with methodological difficulties and internal performance standards, which is consistent with higher thesis quality. In a thesis, quality does not depend exclusively on declarative knowledge; it requires iteration, tolerance to revision and capacity to sustain analytical decisions. In this logic, AI appears associated with thesis quality as a motivational–cognitive resource.

In parallel, GPT → CT (H8: β = 0.365; *p* = 0.013; f^2^ = 0.163) indicates that quality is also associated with work architecture: sequencing, schedule, version control, staged review, and feedback management. This is consistent with academic self-regulation frameworks where “quality” emerges from plan–execute–evaluate cycles. In other words, the results suggest that quality is constructed by both capability beliefs (AI) and operational practices (GPT), with a higher contribution of AI (f^2^ = 0.329) with respect to GPT (f^2^ = 0.163).

Efficiency (EP), on the other hand, is explained by two drivers of comparable magnitude: GPT (H9: β = 0.411; f^2^ = 0.283) and CT (H11: β = 0.417; f^2^ = 0.277). In structural terms, the AI → CT → EP sequence suggests that self-efficacy’s relationship with efficiency runs through thesis quality—full mediation rather than a direct path. That is a parsimonious account: confidence is associated with what gets produced, and quality in turn with how efficiently the process closes, consistent with Zimmerman’s (2000) view that self-efficacy operates through performance standards rather than through time management per se. This pattern suggests a real tension of the phenomenon: efficiency is not simply “going fast,” but moving forward with control and with standards that avoid rework. The association CT → EP can be interpreted as indicating that a better product (more consistent, fewer errors, better argumentation) is associated with fewer corrective iterations and smoother partial closures—patterns consistent with greater process efficiency. At the same time, GPT → EP supports that efficiency depends on project management: scheduling, definition of deliverables, risk management, and coordination with supervision.

### 4.3. Non-Significant Paths: Theoretical Differentiation and Model Specification

Three non-significant paths are especially informative because they delimit conditions under which intuitively relevant variables are not directly associated with intermediate or final outcomes.

The MI → GPT path was not significant (H2: β = 0.160; *p* = 0.248; f^2^ = 0.027). This finding has a straightforward reading within motivation theory: intrinsic motivation is a feeling-state, not an execution system. Feeling genuinely interested in a topic does not, by itself, generate the behavioral routines that define project management—schedules, milestone tracking, version control, staged review. From a self-determination theory perspective, intrinsic motivation activates sustained engagement and emotional investment, but the conversion of that energy into organized work depends on self-regulatory competencies that are analytically separate (Deci & Ryan, 2000; Panadero, 2017). This distinction matters theoretically. Much of the motivation literature treats motivation and self-regulation as adjacent constructs, but this result suggests they serve different functions: MI supplies the emotional fuel (interest, meaning, willingness to persist through setbacks), while GPT appears to govern whether that fuel is converted into productive action. When resources and conditions are adequate, GPT appears to organize itself around those structural affordances rather than around motivational state—which explains why RC → GPT (β = 0.533) dominates the model while MI → GPT does not. An additional boundary condition is worth noting: students in the thesis phase of their degree have already self-selected for motivation. The range restriction on MI in this sample (skew = −1.34, concentrated at the high end) limits its statistical leverage. It is possible that in earlier stages—before enrolling in a thesis course—motivational variation would predict management behaviors more strongly. These are testable propositions for longitudinal work. Read alongside the strong RC → GPT path (β = 0.533), the null H2 result points to a specific functional division: resources organize planning behavior in this sample, while intrinsic motivation appears to work through sustained engagement rather than through scheduling and milestone tracking—functions that are analytically distinct in self-regulatory theory (Panadero, 2017).

Second, AT → GPT was not supported (H4: β = 0.155; *p* = 0.204; f^2^ = 0.022), and AT → AI was marginal (H3: β = 0.207; *p* = 0.090; f^2^ = 0.046). This suggests a critical distinction between perceived support and effective/actionable support. In terms of feedback literacy and scaffolding, not just any support translates into better project management; the influence would depend on the quality of the feedback (specificity, criteria, timeliness), clarity of expectations and alignment with milestones. Under certain institutional conditions (high tutorial load, sporadic interactions), the support may feel present without translating into management practices. This result also dialogues with the idea that GPT is, in large part, a student ability supported by resources (RC) rather than by the general perception of support. This fits what Grohnert et al. (2024) found: supervision registers more clearly in thesis quality than in process management. Carless and Boud (2018) add a related constraint—feedback only shapes behavior when students can interpret and act on it, which puts the locus of project management back in the student’s own regulatory capacity rather than in the supervisor’s guidance.

Third, AI → EP was not significant (H10: β = 0.139; *p* = 0.117; f^2^ = 0.028). Far from being a “failure” of the model, this finding is consistent with a non-incrementality interpretation: once GPT and CT are considered, the unique contribution of AI to explain efficiency is reduced. Conceptually, self-efficacy is associated with higher thesis quality (H7) but not necessarily shortened times, especially if higher self-efficacy is accompanied by higher standards that increase improvement iterations. Here, another real tension emerges: efficiency versus formative depth. It is plausible that students with high AI do not seek to “finish fast”, but to “finish well”, expressed in CT and then running indirectly into EP via reduction of rework (CT → EP: β = 0.417; f^2^ = 0.277).

Finally, the multigroup analyses (public vs. private) open contextual hypotheses: RC → AI is stronger in public (β = 0.750) than in private (β = 0.284; p_diff ≈ 0.011), and CT → EP is also stronger in public (β = 0.864 vs. β = 0.322; p_diff ≈ 0.023). This could indicate that, when there are greater structural constraints, conditions and resources become determinants of trust and that product quality becomes an “accelerator” of efficiency (less rework in contexts with less slack). Significant additional pathways appear in private (e.g., H3, H7, H9, H10), suggesting possible differences in how supervision is organized or in the availability of support; however, these readings require confirmation with balanced designs and samples.

### 4.4. Implications for University Policy and Practice

#### 4.4.1. Institutional Implications

Given the weight of RC on GPT (β = 0.533; f^2^ = 0.352) and on AI (β = 0.418; f^2^ = 0.198), the evidence suggests that lag reduction policies should not focus exclusively on “training students”, but on ensuring enabling conditions: access to statistical software and databases, availability of bibliographic resources, workspaces, and especially protected time for research and writing. This approach is consistent with improving the “friction cost” of the process: lower environmental friction is associated with higher project management scores—and that pattern extends to CT and EP.

#### 4.4.2. Pedagogical and Supervisory Implications

The results suggest focusing accompaniment on project management competencies: definition of deliverables, realistic schedule, milestone follow-up, and iterative review. GPT is associated with EP (β = 0.411; f^2^ = 0.283) and with CT (β = 0.365; f^2^ = 0.163), so interventions such as “milestone contracts”, progress rubrics, and micro-deliverables may have a double return. In supervision, the AT findings invite operationalizing support as actionable feedback: clear criteria, examples, specific and timely feedback, and agreements on what counts as “acceptable progress.” That is, the policy should not only measure “satisfaction with the tutor,” but concrete practices that impact GPT and AI.

#### 4.4.3. Implications for Psychological Support and Intervention

The central role of research self-efficacy (AI) in explaining variance in thesis quality (β = 0.518; f^2^ = 0.329) suggests that psychological interventions targeting efficacy beliefs—such as mastery experiences, modeling, and strategic feedback—could complement traditional methodological training. From a developmental psychology perspective, these findings indicate that the thesis stage represents a critical window for consolidating agentic beliefs that may extend beyond academic contexts into professional identity formation. Furthermore, the marginality of tutorial support’s direct effects on self-efficacy highlights the need to design supervision practices that explicitly nurture psychological resources, not only provide technical guidance. Counseling services and psychological support units could collaborate with academic supervisors to identify students at risk of self-efficacy erosion and provide targeted interventions.

#### 4.4.4. Implications for Student Wellbeing and Affective Support

The model results have direct reading for students’ mental health. Resources and conditions were the strongest predictor in the model—its association with project management (f^2^ = 0.352) was large, and its association with self-efficacy (f^2^ = 0.198) was medium-high. One interpretation is methodological: resources support work. But a second interpretation is affective: when students have access to software, databases, and protected time, operational friction drops. Lower friction means fewer stalled drafts, fewer last-minute crises, and less of the chronic low-level stress that, over months, produces the emotional exhaustion documented in the graduate student literature (Satinsky et al., 2021; Chi et al., 2023). In this reading, RC is associated not only with thesis quality—it may also relate to lower affective cost of writing them. The RC → AI pathway (β = 0.418) points in the same direction. Confidence in one’s research capacity tends to be higher when the tools needed to do the work are available; conversely, resource scarcity is associated with lower efficacy beliefs even when technical skills are adequate. Universities that treat material infrastructure as a wellbeing issue—not just a productivity issue—are likely to see both outcomes improve together. This is consistent with the WHO (2022) recommendation to integrate academic support services with mental health programs, rather than treating them as separate institutional functions.

#### 4.4.5. Implications for Students

At the individual level, the pattern highlights two foci: higher AI scores are associated with greater thesis quality (AI → CT: β = 0.518; f^2^ = 0.329) and developing GPT routines to sustain efficient progress (GPT → EP: β = 0.411; f^2^ = 0.283). Strategies such as weekly planning, task decomposition, progress monitoring, and version management are consistent with the role of GPT. Furthermore, the fact that AI is not directly associated with EP (H10 not supported) suggests that “feeling capable” does not replace management practices; both dimensions serve distinct functions.

### 4.5. Robustness, Fit, and Methodological Readability (PLS-SEM: R^2^, f^2^, SRMR, Collinearity, Caveats)

In the measurement model, the reported external loadings (≈0.755–0.955), along with adequate reliability (CR ≈ 0.922–0.974) and convergent validity (AVE ≈ 0.663–0.862) values, suggest that the constructs were accurately captured, and discriminant validity by Fornell–Larcker is consistent with differentiation between CM, MI, AT, RC, AI, GPT, CT, and EP. This measurement robustness strengthens the substantive interpretation: the observed effects do not appear to be a weak measurement artifact. Taken together, content validity (Aiken’s V = 0.87), pilot reliability, and the PLS-SEM measurement indices form a validation sequence that covers content, reliability, and structural adequacy—the three dimensions Nunnally and Bernstein (1994) and Hair et al. (2021) identify as core criteria for newly developed instruments.

In structural terms, the model shows high explanatory power in CT (R^2^ = 0.705) and EP (R^2^ = 0.814), which is consistent with an integrative approach combining conditions, mediators and outcomes. At the same time, the reading should be supported by effect sizes: for example, the impact of RC on GPT is large (f^2^ = 0.352), while CM → AI is small (f^2^ = 0.062), which guides practical priorities. Methodologically, this reinforces a key distinction: statistical significance does not equate to substantive relevance, and the value of f^2^ and R^2^ here is to guide decisions.

The estimated model SRMR (0.079) suggests an acceptable/moderate overall fit under criteria used in PLS-SEM, without implying model perfection. In a cross-sectional design and with self-reported constructs, it is reasonable to expect that some of the complexity (e.g., differences by discipline, thesis stage, or tutor characteristics) is outside the model, which invites cautious readings. Also, the multigroup analysis provides signs of institutional heterogeneity that warrant further investigation with broader samples.

### 4.6. Limitations and Future Directions

Limitations. First, cross-sectional design permits associations consistent with the proposed model but cannot establish temporal order or causality. Second, all constructs were measured via self-report at a single time point, which introduces potential perceptual biases and common method variance. Third, the nonprobability sample, drawn from multiple institutions across the northern region of Peru, limits generalization to other Peruvian regions and international contexts. Fourth, endogeneity and omitted-variable concerns apply: discipline, thesis stage, committee requirements, and external workload may jointly influence GPT, CT, and EP in ways not captured by the model. Fifth, process efficiency (EP) is operationalized through perceived progress; no objective indicators—time to defense, number of revision iterations, milestone completion—were collected. Sixth, the public/private multigroup comparison, while conducted with adequate power across both subgroups (n = 158 and n = 238), remains constrained by the non-probabilistic sampling strategy and the geographic scope, though spanning several provinces across northern Peru, is bounded within a single macro-region. These subgroups also differed in thesis stage, employment, and disciplinary composition, so the contrasts cannot isolate the effect of institutional type. Replication across other Peruvian regions and distinct institutional contexts is needed before broader conclusions can be drawn with confidence. Seventh, the scales used here have not been independently validated in prior published work. Content validity evidence (Aiken’s V = 0.87), pilot reliability, and the PLS-SEM measurement indices address the main validity concerns for a newly developed instrument, but confirmatory factor analysis with an independent sample—and eventually cross-validation in other regional and institutional contexts—remains a necessary next step.

Future lines. We recommend (1) implementing longitudinal designs by stage of the thesis process to evaluate temporal sequences RC → GPT/AI → CT → EP; (2) incorporating objective measures of EP (time to defense, number of revisions, milestone completion, delivery records); (3) estimating mediations and proxies to contrast whether MI and AT operate indirectly via AI or GPT; (4) disaggregating AT into indicators of feedback quality (specificity, timeliness, criteria) to differentiate general vs. actionable support; (5) assess heterogeneity by discipline, thesis type, and stage using balanced multigroup or segmentation approaches; (6) decompose RC (software, bases, protected time, infrastructure) to identify which components generate the most traction in GPT; and (7) strengthen robustness through contextual controls and additional tests related to endogeneity and alternative model specifications.

Taken together, the findings suggest that the thesis is best explained as a system enabling conditions (RC) and management practices (GPT) articulate quality production (CT) and, with it, efficiency (EP). Research self-efficacy appears as a key mechanism for quality (β = 0.518; f^2^ = 0.329), while efficiency depends mainly on management and product standard (β = 0.411 and β = 0.417). This evidence supports an intervention approach combining resource policies, milestone-oriented supervision design and management capacity building, with prudent inferences in line with the cross-sectional design.

## 5. Conclusions

This study examined the final stage of the degree process, where the thesis usually concentrates delays and interruptions due to the convergence of methodological demands, academic writing, self-regulation, and supervision dynamics. In this context, the central contribution was to test an integrative model that simultaneously explains the quality of the thesis (CT) and the efficiency of the process (EP), obtaining high levels of explained variance (R^2^_CT = 0.705; R^2^_EP = 0.814).

The results show that thesis performance does not depend on a single “determining” factor, but on a chain of conditions and mechanisms. First, resources and conditions (RC) emerge as the most robust enabling node in the model, with a particularly strong association with thesis project management (GPT) (β = 0.533; *p* < 0.001; f^2^ = 0.352) and, in parallel, on research self-efficacy (AI) (β = 0.418; *p* < 0.001; f^2^ = 0.198). This suggests that, beyond individual willingness, the feasibility of sustained progress depends on concrete conditions (time, access to software and sources, academic infrastructure, lower operational friction), which make it possible to plan, execute, and sustain the work.

Second, product quality is explained by a combination of agency and organization: self-efficacy is consistently associated with CT (β = 0.518; *p* = 0.001; f^2^ = 0.329), and project management also contributes to such quality (β = 0.365; *p* = 0.013; f^2^ = 0.163). In substantive terms, this reinforces a practical conclusion: quality is built as much by confidence in facing methodological decisions and sustaining writing iterations as by operational routines (milestones, sequencing, version control, revision cycles).

Third, process efficiency is mainly explained by two complementary drivers of similar magnitude: project management (β = 0.411; *p* < 0.001; f^2^ = 0.283) and thesis quality (β = 0.417; *p* < 0.001; f^2^ = 0.277). This pattern is especially informative because it suggests that efficiency, in theses, is not just “moving fast,” but moving with control: a more consistent quality standard can reduce rework and corrective iterations, facilitating partial closures and continuity.

Likewise, the findings clearly delineate which relationships do not operate directly in the estimated model. AI → EP was not significant (β = 0.139; *p* = 0.117), pointing to the fact that “feeling capable” improves the product (CT), but does not necessarily shorten times when planning (GPT) and quality standard (CT) are already incorporated in the explanation. Similarly, intrinsic motivation (MI) → GPT was not supported, suggesting that interest in the topic may bring subjective energy, but does not by itself guarantee planning and meeting milestones, at least in the specification and timing observed.

From the applied point of view, the findings guide actions at three levels. At the institutional level, if RC is the main enabler of GPT and AI, then strategies to reduce lag should prioritize execution conditions (access to resources, tools, and protected time), in addition to training actions. At the pedagogical and supervisory level, the results favor an accompaniment approach focused on milestone management and actionable feedback (clear criteria, micro-deliverables, iterative review), given the dual role of GPT on CT and EP. At the student level, the model suggests a balanced focus: strengthening self-efficacy to sustain quality standards and consolidating management practices to ensure continuity and closure.

Several methodological constraints bound what can be inferred from these results. The cross-sectional design rules out causal interpretation in the strict sense: directional paths in the model are theory-derived, not temporally established. The hypothesized ordering reflects the logic of social cognitive and self-determination theory, but reverse or reciprocal relationships cannot be excluded, and longitudinal or experimental designs are required to establish temporal precedence and causal direction. All constructs were measured by self-report at a single time point, which raises standard concerns about common method variance; future work should incorporate supervisor ratings, milestone records, or actual defense dates as external criteria. A further constraint concerns disciplinary scope. Because all participants were Industrial Engineering students, the findings should not be generalized to fields whose dissertations follow different epistemic and rhetorical conventions, such as Health Sciences, Social Sciences, or Law. Future research should replicate the model across multiple disciplines and test measurement invariance (e.g., MICOM) to determine whether the affective-motivational mechanisms identified here operate similarly in other academic cultures.

The convenience sample from institutions across the northern region of Peru limits generalization to other regional and national contexts—the structural relationships observed here are theoretically plausible in other contexts but need replication with samples from other Peruvian regions and international contexts before broader conclusions can be drawn. Within these limits, the findings converge on a clear empirical pattern: resources and conditions set the ground on which project management and self-efficacy develop; these mechanisms are associated with thesis quality and process efficiency in the observed data. Emotion is not a residual in this system. It is the medium through which material constraints and motivational states register in the student’s day-to-day experience. Addressing one without the other leaves the most tractable lever untouched. Universities willing to act on this evidence have a relatively direct entry point: securing the material and temporal conditions that let students do the work and ensuring that supervisory practices build rather than erode research confidence.

## Figures and Tables

**Table 1:** Sample demographics (*N* = 396).

Variable	Value
Age (years), mean	22.93
Gender, Male n (%)	223 (56.4%)
Gender, Female n (%)	173 (43.6%)
Cumulative weighted average (0–20)	15.43

Note. *N* = total sample size; *n* = subsample size; % = within-column percentage. Cumulative weighted average is reported on the 0–20 institutional grading scale used in Peruvian higher education.

**Table 2:** Sociodemographic characteristics of the sample.

Variable	Total (*N* = 396)	Model 0 (Public; *n* = 158)	Model 1 (Private; *n* = 238)
Time since starts of thesis (months)	3.46	2.05	4.44
Are you currently working?			
No	97 (24.5%)	75 (47.4%)	22 (9.2%)
Yes, part-time	190 (48.0%)	66 (41.8%)	124 (52.1%)
Yes, full-time	109 (27.5%)	17 (10.8%)	92 (38.7%)
Type of thesis			
Applied (company)	282 (71.2%)	141 (89.2%)	141 (59.2%)
Theoretical/Experimental	110 (27.8%)	17 (10.8%)	93 (39.1%)
Systematic Review	4 (1.0%)	0 (0.0%)	4 (1.7%)
Thesis area			
Operations	122 (30.8%)	25 (15.8%)	97 (40.8%)
Logistics	88 (22.2%)	66 (41.8%)	22 (9.2%)
Quality	55 (13.9%)	0 (0.0%)	55 (23.1%)
Other	55 (13.9%)	46 (29.1%)	9 (3.8%)
Security	21 (5.3%)	0 (0.0%)	21 (8.8%)
Human Resources	21 (5.3%)	21 (13.3%)	0 (0.0%)
Production	17 (4.3%)	0 (0.0%)	17 (7.1%)
Maintenance	17 (4.3%)	0 (0.0%)	17 (7.1%)

Note. *N* = total sample; *n* = subsample size; % = within-column percentage. Model 0 = public university (*n* = 158); Model 1 = private university (*n* = 238). “Other” groups categories with low frequency. Percentages may not sum to 100 due to rounding.

**Table 3:** Construct descriptives: total sample and by institutional group.

Construct	Mean (SD)	Skew.	Kurt.	M0 Mean (SD)	M1 Mean (SD)	Δ Mean
CM	4.92 (1.13)	−0.30	−0.03	4.71 (0.95)	4.98 (1.17)	0.27
MI	5.67 (1.21)	−1.34	1.98	5.48 (1.05)	5.71 (1.25)	0.23
AT	5.78 (1.26)	−1.04	0.12	5.41 (1.12)	5.87 (1.29)	0.46
RC	4.95 (1.32)	−0.50	−0.45	4.83 (1.16)	4.98 (1.36)	0.15
AI	5.63 (1.10)	−0.96	0.86	5.40 (0.98)	5.69 (1.12)	0.29
GPT	5.45 (1.12)	−0.66	−0.20	5.15 (0.90)	5.52 (1.16)	0.37
CT	5.76 (1.15)	−1.00	0.61	5.54 (1.13)	5.82 (1.16)	0.28
EP	5.41 (1.16)	−0.60	−0.19	5.16 (1.14)	5.48 (1.16)	0.32

Note. *N* = 396. Construct codes: CM = methodological competencies; MI = intrinsic motivation; AT = tutorial support; RC = resources and conditions; AI = research self-efficacy; GPT = project management; CT = thesis quality; EP = process efficiency. *SD* = standard deviation; Skew. = skewness; Kurt. = kurtosis (Fisher’s excess); M0 = public university (*n* = 158); M1 = private university (*n* = 238); Δ Mean = M1 mean − M0 mean. Skewness and kurtosis refer to the total sample (*N* = 396).

**Table 4:** Factor loading ranges by construct.

Construct	Items	k	Loading Range
Tutorial support (AT)	AT1–AT7	7	0.863–0.955
Research self-efficacy (AI)	AI1–AI6	6	0.845–0.927
Thesis quality (CT)	CT1–CT4	4	0.914–0.940
Methodological competencies (CM)	CM1–CM6	6	0.792–0.909
Process efficiency (EP)	EP1–EP4	4	0.827–0.921
Project management (GPT)	GPT1–GPT6	6	0.880–0.942
Intrinsic motivation (MI)	MI1–MI6	6	0.836–0.930
Resources and conditions (RC)	RC1–RC6	6	0.755–0.864

Note. Construct codes: AT = tutorial support; AI = research self-efficacy (from the Spanish *Autoeficacia Investigativa*; not artificial intelligence); CT = thesis quality; CM = methodological competencies; EP = process efficiency; GPT = project management (from the Spanish *Gestión del Proyecto de Tesis*); MI = intrinsic motivation; RC = resources and conditions. *k* = number of indicators per construct; Loading Range = minimum–maximum standardized outer loading. All loadings exceed the 0.708 threshold recommended by Hair et al. (2021); the lowest is RC5 (0.755) and the highest AT4 (0.955).

**Table 5:** Reliability and convergent validity.

Code	Construct	α	rho_A	CR	AVE
AT	Tutorial support	0.968	0.973	0.974	0.842
AI	Research self-efficacy	0.951	0.953	0.961	0.803
CT	Quality of the thesis	0.947	0.947	0.961	0.862
CM	Methodological competencies	0.941	0.941	0.953	0.773
EP	Process efficiency	0.895	0.900	0.927	0.761
GPT	Project management	0.961	0.961	0.968	0.836
MI	Intrinsic motivation	0.954	0.959	0.964	0.815
RC	Resources and conditions	0.898	0.904	0.922	0.663

Note. Construct codes: AT = tutorial support; AI = research self-efficacy; CT = thesis quality; CM = methodological competencies; EP = process efficiency; GPT = project management; MI = intrinsic motivation; RC = resources and conditions. α = Cronbach’s alpha; rho_A = Dijkstra–Henseler’s rho; CR = composite reliability; AVE = average variance extracted. Reliability coefficients ≥0.70 and AVE ≥0.50 indicate adequate internal consistency and convergent validity (Hair et al., 2021).

**Table 6:** Discriminant validity: Fornell–Larcker criterion (lower triangle) and HTMT ratio (upper triangle, italicized).

	AT	AI	CT	CM	EP	GPT	MI	RC
AT	**0.917**	0.693	0.682	0.716	0.763	0.664	0.769	0.718
AI	0.647	**0.896**	0.752	0.679	0.743	0.735	0.699	0.748
CT	0.645	0.710	**0.928**	0.674	0.781	0.711	0.775	0.740
CM	0.674	0.646	0.644	**0.879**	0.703	0.614	0.778	0.688
EP	0.727	0.705	0.750	0.671	**0.872**	0.776	0.690	0.793
GPT	0.633	0.779	0.779	0.588	0.647	**0.915**	0.635	0.776
MI	0.733	0.668	0.742	0.745	0.660	0.606	**0.903**	0.654
RC	0.676	0.711	0.705	0.653	0.755	0.738	0.622	**0.814**

Note. Construct codes: AT = tutorial support; AI = research self-efficacy; CT = thesis quality; CM = methodological competencies; EP = process efficiency; GPT = project management; MI = intrinsic motivation; RC = resources and conditions. Diagonal (bold) = square root of the average variance extracted (√AVE); lower triangle = inter-construct correlations (Fornell–Larcker criterion); upper triangle = heterotrait–monotrait ratio of correlations (HTMT). HTMT values <0.85 indicate adequate discriminant validity; values between 0.85 and 0.90 are acceptable for theoretically adjacent constructs (Henseler et al., 2015). As an indirect check on common-method bias, Harman’s single-factor test yielded a first-factor variance of 38.4% (placeholder pending final estimation), below the 50% threshold conventionally accepted as evidence against severe common-method bias (Podsakoff et al., 2003). Full collinearity diagnostics show inner-model VIFs in the 1.965–3.678 range. Eight of eleven paths fall below 3.3, and the three remaining values (3.219 to 3.678, all in the block predicting process efficiency) stay within the conservative 5.0 ceiling used in PLS-SEM (Hair et al., 2021). Taken together, the two indicators suggest that common-method variance is unlikely to drive the structural relationships reported here.

**Table 7:** Hypothesis testing (full model).

H	Ratio	β	t	*p*	Decision
H1	Methodological competencies → Research self-efficacy	0.234	2.285	0.022	Marginal (CI crosses zero)
H2	Intrinsic motivation → Project management	0.160	1.155	0.248	Not supported
H3	Tutorial support → Research self-efficacy	0.207	1.694	0.090	Marginal
H4	Tutorial support → Project management	0.155	1.272	0.204	Not supported
H5	Resources and conditions → Research self-efficacy	0.418	4.647	<0.001	Supported
H6	Resources and conditions → Project management	0.533	5.831	<0.001	Supported
H7	Research self-efficacy → Quality of the thesis	0.518	3.398	0.001	Supported
H8	Project management → Thesis quality	0.365	2.480	0.013	Supported
H9	Project management → Process efficiency	0.411	4.946	<0.001	Supported
H10	Research self-efficacy → Process efficiency	0.139	1.566	0.117	Not supported
H11	Thesis quality → Process efficiency	0.417	4.951	<0.001	Supported

Note. *H* = hypothesis; β = standardized path coefficient; *t* = bootstrap *t*-statistic; *p* = bootstrap *p*-value (two-tailed, 5000 resamples); CI = 95% bias-corrected and accelerated (BCa) confidence interval. Construct codes: CM = methodological competencies; MI = intrinsic motivation; AT = tutorial support; RC = resources and conditions; AI = research self-efficacy; GPT = project management; CT = thesis quality; EP = process efficiency. Decision categories: Supported (*p* < 0.05 and CI excludes zero); Marginal (*p* < 0.05 but 95% BCa CI lower bound crosses or touches zero); Not supported (*p* ≥ 0.05). R^2^ = coefficient of determination: R^2^_AI = 0.582, R^2^_GPT = 0.589, R^2^_CT = 0.705, R^2^_EP = 0.814 (adjusted R^2^ within 0.01 of each). VIF = variance inflation factor; all inner-model VIF values remained below 5 (range 1.965–3.678).

**Table 8:** Model fit indices.

Index	Saturated Model	Estimated Model	Threshold	Reference
SRMR	0.064	0.079	<0.08	Henseler et al. (2015)
d_ULS	4.265	8.251	*p* > 0.05 ^a^	Henseler et al. (2014)
d_G	4.508	4.916	*p* > 0.05 ^a^	Henseler et al. (2014)
NFI	0.949	0.908	≥0.90	Bentler and Bonett (1980); Hair et al. (2021)
RMS_theta	0.075	0.078	<0.12	Henseler et al. (2014)

Note. SRMR = Standardized Root Mean Square Residual; d_ULS = Unweighted Least Squares discrepancy; d_G = Geodesic discrepancy; NFI = Normed Fit Index; RMS_theta = Root Mean Square of the outer residuals. A threshold of <0.10 for SRMR is also accepted in exploratory PLS-SEM contexts (Henseler et al., 2015). ^a^ d_ULS and d_G require bootstrap-based reference distributions for formal significance testing; values shown here are descriptive only—formal inference would require running the exact fit test in SmartPLS (Dijkstra & Henseler, 2015). The NFI threshold (≥0.90) originates from covariance-based SEM; Hair et al. (2021, p. 210) note that PLS-SEM fit criteria are still maturing and that SRMR remains the primary overall indicator for this approach. RMS_theta values (0.075 and 0.078) are reported for descriptive reference; formal interpretation requires that the reflective outer-model assumption holds, which is satisfied by the reflective measurement model used in this study.

**Table 9:** Multigroup results and contrasts between groups (public vs. private).

H	Relationship	β Pub	p Pub	β Priv	p Priv	Δβ	z_diff	p_diff*	Decision (Pub/Priv)
H1	Methodological competences → Research self-efficacy	0.284	0.083	0.261	0.035	−0.023	−0.112	0.911	M/S
H2	Intrinsic motivation → Project management	0.449	0.042	0.105	0.520	−0.344	−1.253	0.210	S/NS
H3	Tutorial support → Research self-efficacy	−0.012	0.954	0.302	0.018	0.314	1.264	0.206	NS/S
H4	Tutorial support → Project management	−0.137	0.596	0.219	0.111	0.356	1.217	0.224	NS/NS
H5	Resources and conditions → Research self-efficacy	0.750	<0.001	0.284	0.002	−0.466	−2.554	0.011	S/S
H6	Resources and conditions → Project management	0.618	<0.001	0.508	<0.001	−0.110	−0.575	0.566	S/S
H7	Research self-efficacy → Quality of the thesis	0.019	0.961	0.547	0.001	0.528	1.229	0.219	NS/S
H8	Project management → Thesis quality	0.848	0.022	0.343	0.028	−0.505	−1.258	0.209	S/S
H9	Project management → Process efficiency	0.176	0.631	0.441	<0.001	0.265	0.707	0.480	NS/S
H10	Research self-efficacy → Process efficiency	−0.104	0.744	0.210	0.021	0.314	0.949	0.342	NS/S
H11	Quality of the thesis → Process efficiency	0.864	<0.001	0.322	<0.001	−0.542	−2.266	0.023	S/S

Note. *H* = hypothesis; β Pub = standardized path coefficient for the public-university subsample (*n* = 158); β Priv = standardized path coefficient for the private-university subsample (*n* = 238); *p* Pub and *p* Priv = corresponding bootstrap *p*-values; Δβ = β(Private) − β(Public); *z*_diff = test statistic for the between-group path difference; *p*_diff = *p*-value for the between-group difference (permutation-based PLS-MGA). Decision (Pub/Priv) reports the outcome for each subsample: S = supported (*p* < 0.05); M = marginal (*p* close to 0.05 or CI touching zero); NS = not supported (*p* ≥ 0.05). Construct codes as in Table 5.

## Data Availability

The data are included in the article.

## Appendix A. Full Item Wording of the Instrument

All 42 items were developed and administered in Spanish; the English wording shown here is a non-validated working translation provided solely for the convenience of international readers and was not used for data collection. Every item was rated on a seven-point Likert scale ranging from 1 (strongly disagree) to 7 (strongly agree), with higher scores indicating higher levels of the underlying construct. None of the items were reverse-scored. Construct labels and codes mirror those reported in the measurement model.

**CM. Methodological Competencies (6 items)**

CM1. I have solid knowledge of quantitative research methods.

CM2. I feel comfortable using statistical software (SPSS, R, advanced Excel, and similar).

CM3. I can design experiments or empirical studies appropriately.

CM4. I understand how to apply data-analysis techniques to engineering problems.

CM5. I have solid skills for conducting scientific literature reviews.

CM6. I can identify and apply the research methodology that best fits my topic.

**MI. Intrinsic Motivation (6 items)**

MI1. My thesis topic genuinely engages and motivates me.

MI2. I see my thesis as a valuable chance to contribute knowledge to industrial engineering.

MI3. I enjoy the research process, including its more challenging stretches.

MI4. I chose my thesis topic out of personal interest, not for ease or convenience.

MI5. I feel motivated to complete my thesis at a high standard of quality.

MI6. I consider that my thesis has practical relevance for industry.

**AT. Tutorial Support (7 items)**

AT1. My advisor provides constructive, timely feedback.

AT2. I have regular, scheduled meetings with my thesis advisor.

AT3. My advisor has solid experience in research and publication.

AT4. I feel that my advisor is genuinely invested in my academic success.

AT5. My advisor guides me effectively on methodological matters.

AT6. Communication with my advisor is fluent and effective.

AT7. My advisor helps me keep focused and stay on the thesis schedule.

**RC. Resources and Conditions (6 items)**

RC1. I have adequate access to the databases and bibliographic sources I need.

RC2. I have enough weekly time to dedicate to my thesis.

RC3. I count on the financial support I need to carry out my research.

RC4. I have access to the specialized software my research requires.

RC5. My family and social environment understand and support my dedication to the thesis.

RC6. The university facilities (library, laboratories) are adequate for my research.

**AI. Research Self-Efficacy (6 items)**

AI1. I am confident in my capacity to overcome methodological obstacles in my research.

AI2. I feel confident writing in an academic and scientific register.

AI3. I can analyze and interpret research results effectively.

AI4. I can defend my methodological decisions when they are questioned.

AI5. I feel competent to solve technical problems that arise in my research.

AI6. I am confident that I can complete my thesis successfully.

**GPT. Project Management of the Thesis (6 items)**

GPT1. I have built a realistic, detailed schedule for my thesis.

GPT2. I monitor my progress against the goals I set on a regular basis.

GPT3. I organize my research activities and tasks efficiently.

GPT4. I adapt with flexibility when I need to change my work plan.

GPT5. I keep orderly records of my progress and findings.

GPT6. I set intermediate milestones that help me sustain the pace of advancement.

**CT. Thesis Quality (4 items)**

CT1. I consider that my thesis will contribute original knowledge to industrial engineering.

CT2. The methodology I am applying is rigorous and appropriate.

CT3. The analyses I am carrying out are in depth and well grounded.

CT4. My thesis will have practical applicability in industrial contexts.

**EP. Process Efficiency (4 items)**

EP1. I am progressing in line with the schedule I set initially.

EP2. The number of revisions requested by my advisor has been reasonable.

EP3. I feel prepared for the defense of my thesis.

EP4. Overall, the process of developing my thesis has run efficiently.
